# Substance P signalling in primary motor cortex facilitates motor learning in rats

**DOI:** 10.1371/journal.pone.0189812

**Published:** 2017-12-27

**Authors:** Benjamin Hertler, Jonas Aurel Hosp, Manuel Buitrago Blanco, Andreas Rüdiger Luft

**Affiliations:** 1 Division of Vascular Neurology and Rehabilitation, Department of Neurology, University and University Hospital of Zurich, Zurich, Switzerland; 2 Department of Neurology, University Medical Center Freiburg, Freiburg, Germany; 3 Division of Neurocritical Care, Department of Neurosurgery, University of California Los Angeles, Los Angeles CA, United States of America; 4 cereneo Center for Neurology and Rehabilitation, Vitznau, Switzerland; 5 Department of Neurology, Johns Hopkins University, Baltimore MD, United States of America; Tokai University, JAPAN

## Abstract

Among the genes that are up-regulated in response to a reaching training in rats, Tachykinin 1 (Tac1)—a gene that encodes the neuropeptide Substance P (Sub P)—shows an especially strong expression. Using Real-Time RT-PCR, a detailed time-course of Tac1 expression could be defined: a significant peak occurs 7 hours after training ended at the first and second training session, whereas no up-regulation could be detected at a later time-point (sixth training session). To assess the physiological role of Sub P during movement acquisition, microinjections into the primary motor cortex (M1) contralateral to the trained paw were performed. When Sub P was injected before the first three sessions of a reaching training, effectiveness of motor learning became significantly increased. Injections at a time-point when rats already knew the task (i.e. training session ten and eleven) had no effect on reaching performance. Sub P injections did not influence the improvement of performance within a single training session, but retention of performance between sessions became strengthened at a very early stage (i.e. between baseline-training and first training session). Thus, Sub P facilitates motor learning in the very early phase of skill acquisition by supporting memory consolidation. In line with these findings, learning related expression of the precursor Tac1 occurs at early but not at later time-points during reaching training.

## Introduction

The primary motor cortex (M1) is crucially involved in the storage and encoding of motor memories [1]. During the acquisition of movement sequences in rats, learning-specific regulation of gene expression occurs within M1 [2] following a distinct time-course [3]: among the genes that are up-regulated during the early phase of skill learning, Tachykinin 1 (Tac1) shows an especially large degree of increase when compared to control animals.

Tac1 encodes for the neuropeptides Substance P (Sub P) and Neurokinin A that are expressed through alternative splicing [4]. Sub P is an 11 amino acid peptide that is implicated in numerous biological processes including smooth muscle contraction, inflammation, hypotensive effects and gland secretion [5]. Within the brain, Sub P is released from presynaptic terminals thereby acting as a neurotransmitter. The biological actions of Sub P are mainly mediated by the tachykinin NK1-receptor [6], that is coupled to a 7-transmembrane G-protein (Gq/G11) and regulates the phosphoinositide pathway [5]. NK1-receptors are highly expressed in the caudate putamen, mesencephalon and superior colliculus, but are also present in various other brain regions including M1 [7]. Furthermore, Sub P containing presynaptic terminals were detected in M1, although to a comparably moderate density [8].

In our previous study, up-regulation of Tac1 was detected using a whole genome microarray at single a time-point of early skill acquisition [3] emphasizing a role of Sub P signalling for motor learning. In line with this hypothesis, Sub P promoted learning and memory formation in a series of different learning tasks in rats [9]. Injections into the nucleus basalis magnocellularis or the lateral hypothalamus improved performance in a conditioned place preference test [10, 11]. Furthermore, Sub P facilitated passive avoidance learning when injected either into the globus pallidus or into the amygdaloid body [12]. Inversely, blocking hippocampal Sub P signalling using NK1-receptor antisense oligonucleotides interfered with the acquisition of a Y-maze task [13].

To evaluate the role of Tac1 expression and subsequent Sub P signalling within M1 for motor learning, we quantitatively assessed the temporal dynamic of Tac1 expression at different stages of a reaching task in rats using Real-Time RT-PCR. Furthermore, we injected its substrate Sub P into M1 at different time-points during training and assessed its effect on skill acquisition and memory consolidation.

## Materials and methods

### Animals and experiments

109 adult male Long–Evans rats (8–12 weeks old, raised within our own stock) were used in this study. The Animal Care and Use Committee of the State of Baden-Württemberg (Germany) approved all animal procedures. The rats were randomly assigned to behavioral groups by drawing lots. Trainings were performed at the beginning of the dark phase of a 12 h day/night cycle. For both tasks, exposure to training cage, food, handling and pre-training were identical. Chemicals were purchased from Sigma-Aldrich, unless noted otherwise. Drug application and behavioral testing was performed in a blinded manner and researchers were not aware of group identities.

### Behavioral experiments: motor skill learning

Training sessions were performed at the beginning of the dark phase. Animals were food-restricted for 24 hours before the first pre-training session. During training, animals were kept slightly over their initial weight (300-380g) by providing 50 mg/kg of standard lab diet after each training session. Water was given ad libitum. The skilled-reaching task (SRT) was performed as previously described [14]. The training cage was a 15 x 40 cm chamber (height 30 cm) with a vertical window (1 cm wide, 5 cm high, lower edge 2 cm above ground) in the front wall and a small light sensor in the rear wall (7 cm above ground). The motor task was embedded into an operant conditioning paradigm that involved nose poking a sensor in the rear of the cage to open a sliding door which gave access to the food pellet: rats were first pre-trained for five days to open a motorized sliding door that covered the front window by nose-poking the sensor in the rear. Opening the window gave access to one food pellet (45 mg, Bio-serve, Frenchtown, NJ, USA) located on a pedestal in a distance of 0.5 cm relative to the outside edge of the window. During pre-training pellets were retrieved by tongue. Upon retrieval a pellet dispenser automatically replaced the pellet. The latency between pellet removal and subsequent door opening was measured to assess operant knowledge. In rats that were trained in a skilled-reaching task (i.e. SRT rats), training was initiated by removing the board and placing the pellet on a small vertical pedestal 1.5 cm away from the window. In this position pellets were only retrievable by using the forelimb. Because the diameter of the pedestal was approximately that of the pellet, the pellet was in an unstable position and easily kicked off. During the first 10 door openings (= trials) of the baseline training session, forelimb preference was determined and the pedestal was shifted to one side of the window to allow for reaching with the preferred limb only. To retrieve the pellet, rats had to extend the forelimb towards the target, pronate, open the paw, grasp, and pull the forelimb back while supinating to bring the pellet towards the mouth [15]. Each reaching trial was scored as “successful” (reach, grasp and retrieve) or “unsuccessful” (pellet pushed off pedestal or dropped during retraction). Reaching performance was defined as number of successful trials out of all possible trials (success rate). For the gene-expression experiments, training session consisted of 100 trails or sixty minutes, whichever came first. As a significant Tac1 up-regulation was only present for the initial training sessions, a different design was applied for the pharmacological experiments to slow the skill acquisition process thereby obtaining a higher temporal resolution of the learning curve: at the baseline training, rats were allowed to perform 25 reaching attempts. At the consecutive days, training session consisted of 50 trials/30 minutes. Evolution of reaching performance within a single session was then assessed by sampling the success rate per 5 trials (baseline) or per 10 trials (regular training sessions) in a bin. The intra-session change was calculated by subtracting the percentage of success rate of the first (trial 1–5/10) from the last bin (trial 21-25/41–50). The performance change between two sessions was calculated by subtracting the percentage of success rate of the first bin (trial 1–5/10) of the actual from the last bin (trial 21-25/41–50) of the preceding training session.

For the quantification of Tac1 mRNA, SRT animals were compared to rats subjected to an activity control task (ACT). The ACT group received the same pre-training like SRT rats. The ACT consisted of extending the forelimb through the window to touch a sensor in 1.5cm distance. If the sensor was touched, the investigator gave the rat a pellet directly into the mouth of the rat using forceps. Limb position during reaching in ACT was identical to SRT but no grasping or pellet retrieval was necessary. For the assessment of Tac1 expression, animals were killed after training session 1, 2 and 6 of SRT (n = 42) or ACT (n = 42) - 1h, 7h and 24 h after the training session ended (for SRT and ACT: session 1 - 1h n = 5; -7h n = 4; 24h n = 5; session 2 - 1h n = 5; -7h n = 4; -24h n = 4; session 6 -1h n = 5; -7h n = 5; -24h n = 5). For pharmacological experiments, animals that were assigned to the Sub P group (n = 8) were trained in the SRT for 9 days, whereas rats of the control group (n = 7) were trained for 11 days.

### Tissue and RNA preparation

After decapitation, intact brains were rapidly removed from the skull and the forelimb area of M1 contralateral to the trained forelimb was dissected en-bloc (all cortical layers) at ice temperature according to published coordinates (+2.0–0.0 mm to bregma, 2.0–5.0 mm parasagittal; [16]. All tissue samples were shock frozen in liquid nitrogen and stored at -80°C until use. Tissue was then treated with buffered solution containing mRNase treated, sonicated for homogenization and centrifuged. RNA was isolated using guanidine isothiocyanat (Qiagen, Hilden, Germany), DNase treated and cleaned up (RNeasy Lipid Mini Kit, Qiagen, Hilden, Germany) according to the instructions of the manufacturer. RNA quality was assessed and quantified by UV spectrophotometry. Samples were used only if OD260/280 nm ratio was greater than 1.8. The integrity of each sample was checked on an Agilent Bioanalyzer 2100 (Agilent Technologies, Palo Alto, CA) prior to array processing. cDNA-synthesis and quantitative Real-Time RT-PCR.

### cDNA-synthesis and quantitative real-time RT-PCR

RNA samples were used as a template for cDNA synthesis (SuperScript II RT, Invitrogen, Karlsruhe, Germany) following the manufacturer’s protocol. TaqMan technology (ABI PRISM 7000 Sequence Detection System, Applied Biosystems, Foster City, CA) along with TaqMan Universal PCR MasterMix and TaqMan Gene Expression Assay kits were used for Real-Time RT-PCR. The PCR primer and TaqMan probes were also obtained from Applied Biosystems (TAC1: TaqMan Assay ID Rn00562002_m1). The 5`reporter dye for all probes was FAM and the 3`quencher TAMRA. A passive reference dye (ROX) provided an internal standard for normalization of FAM fluorescence, correcting for fluctuations resulting from volume changes. A total volume of 20 μl PCR reaction mixture containing 9 μl cDNA (or dH_2_O), 1 μl TaqMan probe (250 nmol/l) and primer mix (900 nmol/l, 20x), and 10 μl TaqMan Universal PCR Master Mix (2x) was amplified. Two-step PCR cycling was carried out: first cycle Uracil-N-glycosylase incubation at 50°C for 2 min then at 95°C for 10 min to activate AmpliTaq Gold DNA polymerase, followed by 45 cycles at 95°C for 15 s and 60°C for 1 min. The 18S rRNA was used as housekeeping gene for each target gene. All samples were run in duplicate for the target gene and the housekeeping gene. cDNA was quantified using the “delta-delta Ct" method [17].

### Cannula implantation and injection of drugs

Cannulas for intracortical microinjections were implanted after priming and 3 days before SRT started. Because the preferred paw was not determined at this time-point, guide cannula (34 ga, 15 mm long, Unimed SA, Lausanne, Switzerland) were implanted bilaterally under ketamine (Ketalar, Bayer; 100 mg/kg body weight, i.p.) and xylazine (Streuli; 10 mg/kg body weight, i.p.) anesthesia with the rats fixated in a stereotaxic instrument (Stoelting, Wood Dale, IL USA). Additional ketamine doses (30 mg/kg, i.p.) were administered if necessary. Body temperature was controlled using a heating pad. Buprenorphine (0.01 mg/kg, i.p.) was given after surgery for pain relief. Both guide cannulas were inserted to a depth of 1mm into the center of the forelimb representation of M1 (position relative to bregma: 2.0 mm anterior and 3 mm lateral) and blocked with removable obturators. Cannula became fixed and the scull became closed using dental cement (Flowline, Heraeus Kulzer GmbH, Hanau, Germany). Implants were anchored onto the skull by two screws (2 mm diameter) placed in the frontal and occipital skull. After surgery, animals were returned to their home cages for a 3-day recovery period.

Animals (n = 15) were injected with Sub P at training session one to three, 30 minutes before training started. Drug injections were given only into the cannula contralateral to the trained paw. Rats were sedated briefly with isoflurane (duration < 2 min) and injected with 1 μl of Sub P in saline (Sub P, 1ng/μl, Sigma-Aldrich; n = 8) or with saline alone (SAL, n = 7) using a 5μl microsyringe (Hamilton, Bonaduz, Switzerland) and a motorized pump with the injection speed of 1 μl/min (Stereotaxic microinjection pump, Stoelting, Wood Dale, IL, USA). After each injection, the needle was left in place for 5 min before being slowly retracted. To assess the effect of Sub P on movement execution, 1 μl SP (1ng/μl) was injected in rats of the SAL group at day 10 and 11, a time-point where performance already reached plateau. Furthermore, guide cannula placement was verified histologically.

### Statistical analysis

Statistical analyses and graph presentations were performed using Statistica (version 7; StatSoft, Tulsa, OK, USA) and Prism (version 5; GraphPad Software, La Jolla, CA, USA). Tac1 expression between the SRT and ACT group was compared using independent-samples t-tests, after normal distribution was assessed using the Shapiro-Wilk test. Training performance of SRT animals at training session 1, 2 and 6 were compared using an unpaired Kruskal-Wallis test, as data did not pass the Shapiro-Wilk test for normality. Dunn`s test was performed to correct for multiple comparisons. Learning curves were compared using repeated measures ANOVA, with factors group (Sub P vs. SAL) and time. The sphericity assumption was tested using the Mauchly criterion and the Greenhouse-Geisser correction was used where appropriate. To avoid false-positive results caused by baseline differences, performance at baseline training was added as a covariate. Numerical results are expressed as mean and standard error of the mean (SEM).

## Results

### Expression pattern of Tac1 in response to motor learning

**Fig 1:**
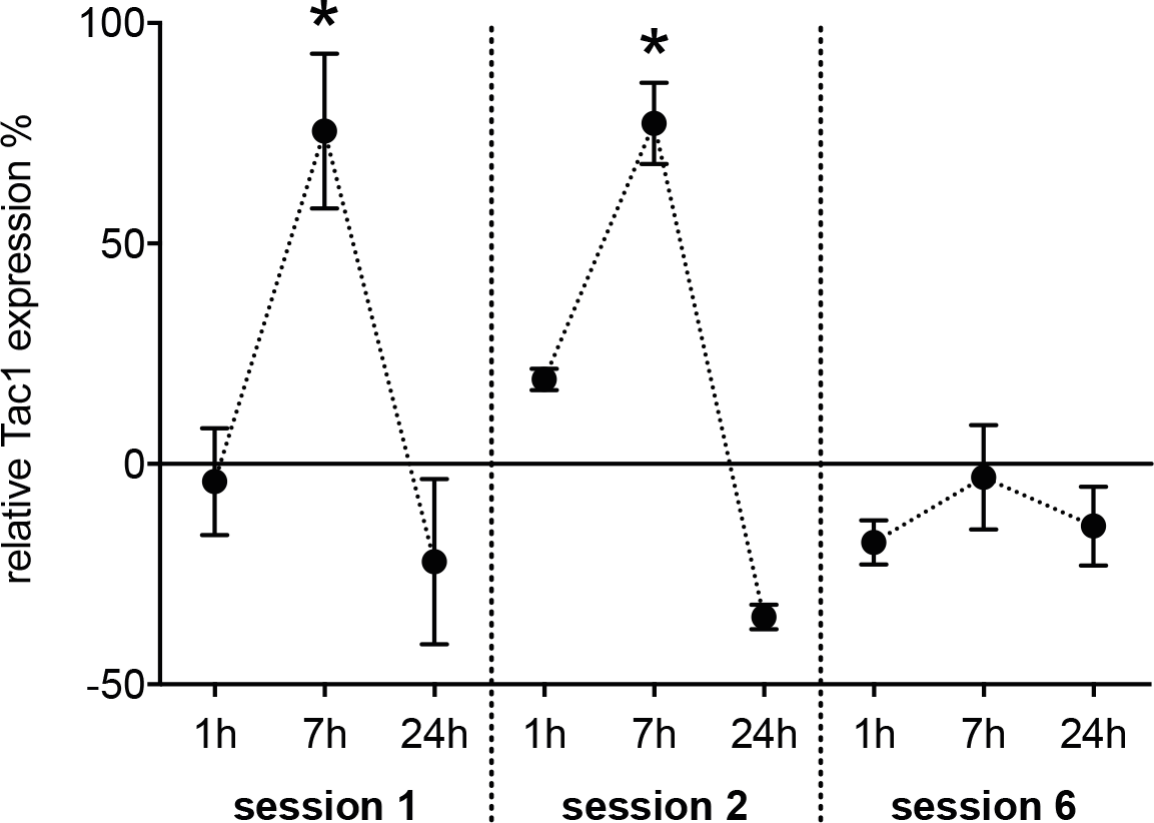
Temporal dynamic of Tac1 expression during motor learning. Levels of Tac1 mRNA within M1 were measured using Real-Time RT-PCR in rats that were trained in a reaching task and related to an activity control at different time points. Tac1 expression was significantly elevated in learning rats seven hours after the training ended in the stage of skill acquisition (training session 1 and 2). This pattern was not observable at a later time-point (training session 6). *: p ≤ 0.05.

Animals in the SRT group showed a significant change in grasping performance between different time points (p = 0.002). Performance of training session 2 (success rate: 0.3 ± 0.03) and 6 (success rate: 0.31 ± 0.04) was significantly higher when compared to session 1 (success rate: 0.21 ± 0.02; p = 0.01 and 0.02 respectively), whereas no difference existed between session 2 and 6 (p = 0.9). This is consistent with a rapid initial movement acquisition of the SRT that is followed by a plateau of performance at later stages [14, 18]. When compared to ACT, Tac1 became significantly up-regulated seven hours after training session 1 (t(df) = 3.9(8); p = 0.005) and 2 (t(df) = 3.2(6); p = 0.019; Fig 1). For all the other time-points, no differences were present between the SRT and ACT group.

### Injection of Substance P into M1 boosts motor learning

**Fig 2:**
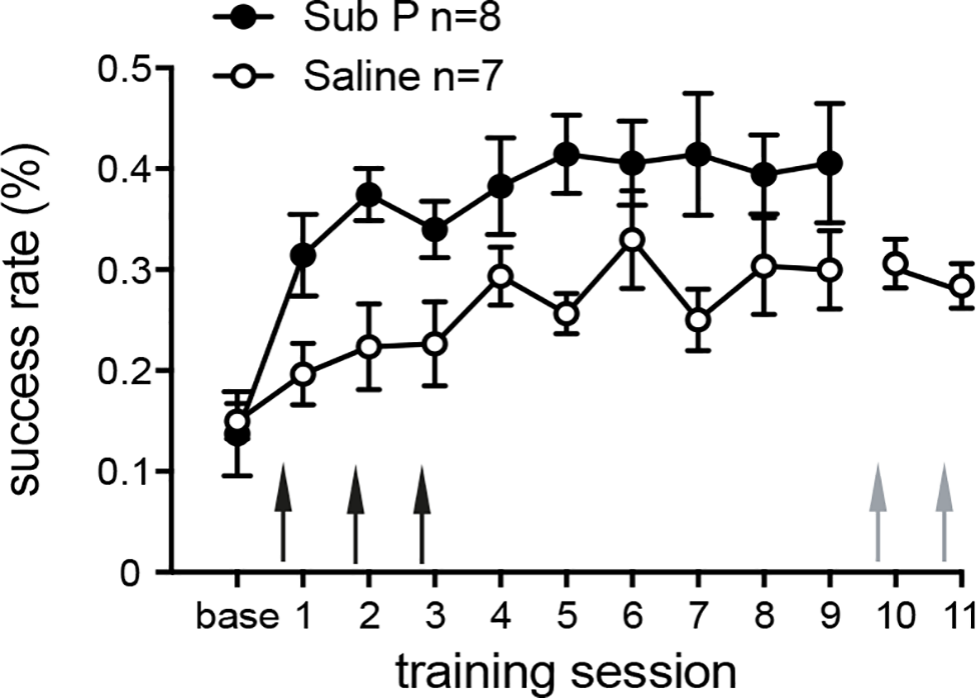
Injection of Substance P boosts motor learning. Injecting 1 μl Sub P into M1 before training session 1–3 (black arrows) significantly improves the performance gain when compared to controls. Injecting the same amount of Substance P to a time-point when animals reached plateau (grey arrows) has no effect. Thus, Substance P affects the acquisition but not execution of a skilled reaching task. Values are presented as mean ± SEM.

Injecting Substance P into M1 during the first three training sessions significantly improved movement acquisition when compared to saline (repeated measures ANOVA, effect of group: F (1,13) = 6.4, p = 0.026; effect of group x time: F (9,117) = 1.2, p = 0.34; Fig 2). Sub P-injections in SAL animals that reached plateau (i.e. session 10 and 11) had no effect on reaching performance consistent with a role for Substance P in skill acquisition but not for execution of already learned skills. There was no effect of Substance P on inter-trial latencies (repeated measures ANOVA, effect of group: F (1,13) = 0.45, p = 0.52; effect of group x time: F (9,117) = 0.38, p = 0.94) and the amount of dropped pellets (repeated measures ANOVA, effect of group: F (1,13) = 0.3; p = 0.58; effect of group x time: F (9,117) = 2.5, p = 0.06).

### Substance P promotes the encoding of motor memory at a very early stage

**Fig 3:**
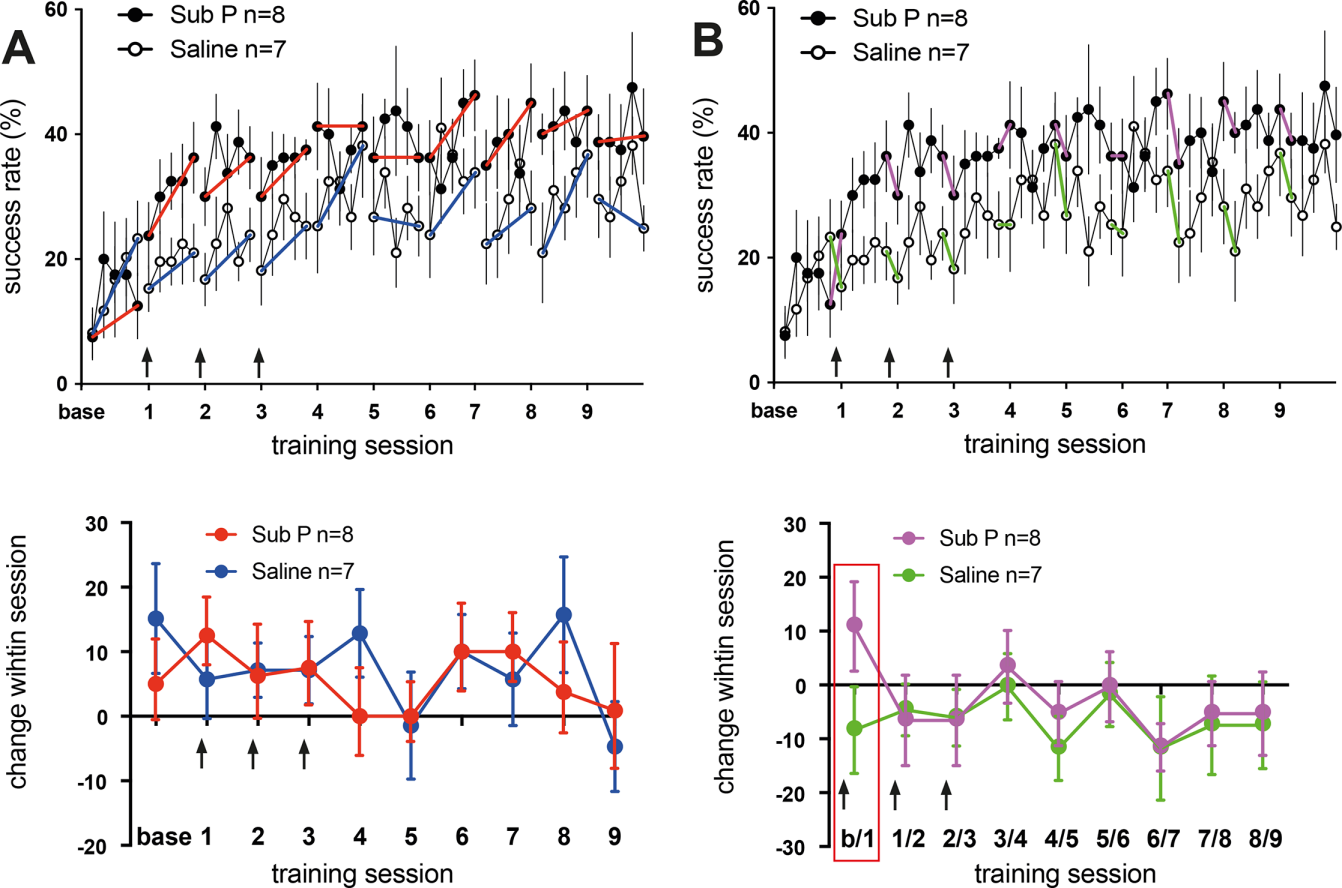
Injection of Substance P augments the encoding of motor memory at a very early stage. (A) top and bottom: to assess intra-session improvement of grasping performance, sessions were divided into quintile-bins. The percentage of successful grasps is plotted per bin. Intra-session improvement is indicated by blue (Saline) or red lines (Sub P). Injection of Sub P did not change intra-session improvement. Values are presented as mean ± SEM. Arrows indicate the time-point of injections. (B) top and bottom: The magnitude of decrease in performance between the end of a training session and the start of the subsequent session (inter-session development) is inversely correlated with the efficacy of motor memory retention. Sub P (filled circles, magenta lines) and Saline animals (open circles, green lines) are characterized by a nearly identical evolution of inter-session development except, the first value (i.e. between base and training session 1, red frame). There, a marked increment in performance occurred in Sub P animals consistent whereas a decrease is present in the Saline group. Values are presented as mean ± SEM. Arrows indicate the time-point of injections.

Sub P-injections did not alter intra-session improvement (Fig 3(A) top). With respect to intra-session change (Fig 3(A) bottom) there was also no difference between groups (repeated measures ANOVA: effect of group: F(1,130) = 0.3; p = 0.57; effect of time: F(9,130) = 0.8; p = 0.62; effect of group x time: F(9,130) = 0.6; p = 0.82). Thus, Sub P had no influence on short-term learning. With respect of performance-change between sessions (Fig 3(B) top and bottom), also no formal significant difference existed between groups (repeated measures ANOVA: effect of group: F(1,117) = 1.2; p = 0.28; effect of time: F(8,117) = 0.8; p = 0.6; effect of group x time: F(8,117) = 0.4; p = 0.9). However, a very strong difference in inter-session change was present between baseline training and the first training session, i.e. after the first injection of Sub P (Sub P: + 11.3 ± 0.08% vs. Saline: - 8 ± 0.08%). Thus, Sub P promotes the encoding of motor memory at very early stages in the learning process without affecting memory consolidation at later time-points.

## Discussion

In response to motor learning, Tac1 expression became significantly up-regulated at early but not at late stages of a skilled reaching training. Likewise, early injections of Sub P into M1 contralateral to the trained paw increased the effectiveness of skill acquisition whereas later injections did not influence the execution of an already learned movement. The improvement in reaching performance within single sessions (i.e. intra-session changes) was not affected by Sub P, consistent with unaltered short-term plasticity.

In response to motor training, profound changes occur within the matrix of M1 at multiple levels [19]. That Sub P improves the effectiveness of skill acquisition could be explained by a facilitative effect on these neuroplastic changes that are thought to form the structural basis of motor learning: (i) At the cellular level, an increase in dendritic arborisation occurs in motoneurons of layer II/III and V [20–22] contralateral to the trained limb. In addition, an initial increase in spine formation is followed by an enhanced turnover that reduces the number of spines to baseline levels while preserving functionally relevant synapses [23]. Intraventricular injections of Sub P lead to both, increase in dendritic arborisation and the formation of novel spines in cerebellar Purkinje cells of rats [24]. Thus, Sub P might support the learning-induced rewiring and formation of novel circuits. (ii) At the level of synaptic weights, motor skill learning induces a long-lasting increase in synaptic strength within M1 horizontal connections of layer II/III that is mediated by long-term potentiation (LTP)-like plasticity [25, 26]. Sub P facilitates the formation of LTP in the hippocampus of guinea pigs [27] and in the visual cortex of rats [28]. LTP critically depends on modulation and trafficking of AMPA-receptors [29]. Sub P inversely modulates AMPA-receptor mediated glutamatergic transmission [30, 31] and leads to induction of AMPA-subunits in dorsal horn neurons in rats [32]. Thus, Sub P may support the formation of long-term plasticity in response to training by modulation of AMPA-receptors. (iii) At the level of gene expression, the transcription factor cFos—that belongs to the group of immediate early genes (IEG)—becomes induced in M1 in response to motor training [33]. Sub P-dependent induction of cFos has been described in the cortex, striatum and hypothalamus of guinea pigs [34] and the trigeminal nucleus of rats [35]. Thus, Sub P could facilitate motor learning by inducing learning-relevant genes in M1. (iiii) At the level of cortical physiology, an increment in M1 excitability occurs in subjects that were trained to play a piano sequence [36]. Sub P has a mainly excitatory effect on cortical neurons [37–39] that is especially prominent in Layer V motoneurons of M1 [38]. Sub P could therefore contribute to an increment of cortical excitability in response to motor skill learning.

Learning-induced expression of Tac1 and effectiveness of Sub P-injections point both to a very early stage in the time-course of motor learning. Learning-induced neuroplastic changes in M1 also follow a defined time-course [19]: with respect to this schedule, Sub P could promote early changes like spine formation, effectiveness of synaptic transmission or changes in cortical excitability. Processes of maturation (increased spine elimination, restoration of the synaptic modification range) or slower and longer-lasting structural changes in the dendritic compartment [40] are less likely to be influenced by Sub P.

In addition to direct effects, Sub P could facilitate neuroplastic changes by modulating dopaminergic (DA) transmission in M1. The primary motor cortex (M1) receives dopaminergic input from a defined neuronal population of the ipsilateral rostral and dorso-lateral ventral tegmental area (VTA) with high target-specificity [41]. The integrity of this projection is essential for successful movement acquisition [42]. Within M1, DA supports learning-related plasticity by inducing cFos [42], allowing LTP formation [43] and by modulating excitability and somatotopy of M1 [44]. Interestingly, key genes of dopaminergic transmission including the main receptor subtypes (i.e. D1- and D2 receptors) become up-regulated in M1 during motor learning in parallel to Tac1 [3]. Sub P is known to support dopaminergic transmission and to increase DA levels in striatum [45, 46], the core of nucleus accumbens and in prefrontal cortex [5, 47]. In addition, D2-receptor expression in frontal cortex of rats becomes modulated by Sub P in reaction of morphine withdrawal [48], leading to changes in receptor density [49].

Although Sub P mainly activates the NK1-receptor [6], effects based on the activation of other NK-receptor subtypes have to be taken into account. Evidence for memory-facilitative effects also exists for the NK3-receptor [50]: systemic administration of the NK3-receptor agonist senktide improves the consolidation of episodic-like memory in rats [51] and fear consolidation depends on NK3-receptor mediated mechanisms within the centromedial amygdala [52]. Furthermore, NK3-R knockout mice show an impaired learning of conditioned avoidance and Morris water maze escape tasks [53]. Even though NK3-receptors are present within layer V of M1 [54], the affinity of Sub P for NK3-receptors is comparably weak (half maximal inhibitory concentration of 67±19 for NK3-receptor vs. 0.19±0.02 for the NK1-receptor [55]). However, a NK3-receptor mediated effect on motor learning cannot be excluded.

In our prior study examining motor learning-related gene expression, Tac1 expression was elevated stronger (5 fold vs. approximately 75%) in SRT vs. controls and the expression peak occurred at a later time point (24h vs. 7h) [3]. However, the control task used in the present study (ACT) had a different design when compared to the previous one: whereas rats were directly rewarded for door openings in the previous version [3], ACT in this study involved the activation of a sensor with the forepaw to obtain food rewards. According to that, the behavioral difference between SRT and ACT is small, as both conditions need the execution of a targeted movement of the forelimb. Furthermore, such a sensor touching may also require learning related plasticity to a certain degree [56]. Thus, differences in the exact timing and degree of Tac1 expression between the previous and the actual study can be easily explained due to this change in control condition.

It has to be also taken into account that the action of Sub P synthesized in response to physiologic Tac1 expression acts through different mechanisms than the injections of Sub P performed here. With respect to the expression profile of Tac1, Sub P concentrations should peak later than 7 hours after the training ended. However, Sub P became injected into M1 30 minutes before the training started. With respect to Fig 3(B, top and bottom), animals that were injected with Sub P started at an elevated level of performance that was maintained through the following training sessions. This phenomenon could be well explained by a facilitated retrieval of skill previously gained in the baseline training session. For hippocampus dependent learning, substantially different mechanisms mediating memory formation or retrieval have been described [57, 58]: the process of memory retrieval—in contrast to memory formation—is independent from protein synthesis, calcium calmodulin kinase (CAMKII)-signalling and NMDA-receptor activation. For motor learning, molecular mechanisms of memory retrieval are not defined so far. Thus, further research is needed to investigate a possible influence of Sub P signaling on retrieval processes in M1.

Apart from motor skill acquisition, other behavioral or situational factors could have influenced Tac1 expression within M1. It is known that Sub P plays a specific role in the behavioral response to stress, such as stress-related addiction or anxiety [59, 60]. Activation of the glucocorticoid receptor—a classic component of the stress response—induces an up-regulation of Tac1 in primary amygdala neurons [59]. Stressful stimuli increase levels of SP and Tac1 mRNA within the amygdala [61] of restrained rats and alters NK1-receptor trafficking in gerbils [62]. Furthermore, stress induces a NK1-recptor mediated activation of neurons in several regions of the stress circuitry including the amygdala, nucleus accumbens and prefrontal cortex [63]. However, neuronal responses to stressful stimuli have been tested in models like elevated platform exposure [61], footshock [63] or immobilization paradigms [62]. In our study, the behavioural difference between the SRT (grasping food-pellet from a pedestal) vs. ACT controls (touching a sensor by paw) hardly explains a relevant difference in stress levels. Additionally, the transition between pre-training and training (i.e. removing the pedestal with food-pellets to encourage grasping attempts) cannot be considered as a stressful stimulus comparable to footshocks or immobilization. Thus, it is very unlikely that different stress levels between groups—or at different stages during motor training—could have confounded our interpretation of results.

Finally it has to be admitted that the behavioral protocols that were applied for gene expression and pharmacological experiments were different with respect to training intensity (100 vs. 50 trials/session). The rationale for this change in paradigms was to slow the skill acquisition process thereby obtaining a higher temporal resolution of the learning curve as Tac1 expression occurred in the early phase of training. Thus, the comparability of both experimental lines is surely limited. However, as intra-session improvement is not affected by Sub P injections and both experimental lines indicate a very early time window for Sub P effects, the differences in protocols should not have influenced the main conclusions of this study: 1. Sub P signaling in M1 facilitates motor learning in the very early stages of skill acquisition. 2. This facilitating effect can be explained by supporting early memory consolidation. 3. In line with these findings, learning related expression of the precursor Tac1 occurs at early but not at later time-points during a reaching training.
